# Prognostic Significance of *VAV3* Gene Variants and Expression in Renal Cell Carcinoma

**DOI:** 10.3390/biomedicines12081694

**Published:** 2024-07-30

**Authors:** Chi-Fen Chang, Bo-Ying Bao, Yu-Mei Hsueh, Pei-Ling Chen, Li-Hsin Chang, Chia-Yang Li, Jiun-Hung Geng, Te-Ling Lu, Chao-Yuan Huang, Shu-Pin Huang

**Affiliations:** 1 Department of Anatomy, School of Medicine, China Medical University, Taichung 406, Taiwan; cfchang@mail.cmu.edu.tw; 2 Department of Pharmacy, China Medical University, Taichung 406, Taiwan; bao@mail.cmu.edu.tw (B.-Y.B.); lutl@mail.cmu.edu.tw (T.-L.L.); 3 Department of Family Medicine, Wan Fang Hospital, Taipei Medical University, Taipei 110, Taiwan; ymhsueh@tmu.edu.tw; 4 Department of Public Health, School of Medicine, College of Medicine, Taipei Medical University, Taipei 110, Taiwan; 5 Department of Urology, National Taiwan University Hospital, College of Medicine, National Taiwan University, Taipei 100, Taiwan; 100117@ntuh.gov.tw; 6 Graduate Institute of Clinical Medicine, College of Medicine, Kaohsiung Medical University, Kaohsiung 807, Taiwan; r120002@kmu.edu.tw; 7 Graduate Institute of Medicine, College of Medicine, Kaohsiung Medical University, Kaohsiung 807, Taiwan; chiayangli@kmu.edu.tw; 8 Department of Medical Research, Kaohsiung Medical University Hospital, Kaohsiung 807, Taiwan; 9 Department of Urology, Kaohsiung Medical University Hospital, Kaohsiung 807, Taiwan; 1000596@kmuh.org.tw; 10 Department of Urology, Kaohsiung Municipal Hsiao-Kang Hospital, Kaohsiung 812, Taiwan; 11 Institute of Medical Science and Technology, College of Medicine, National Sun Yat-Sen University, Kaohsiung 804, Taiwan

**Keywords:** renal cell carcinoma, vav guanine nucleotide exchange factor, genetic variants, gene set enrichment analysis, prognosis

## Abstract

Renal cell carcinoma (RCC) is characterized by high mortality and morbidity rates. Vav guanine nucleotide exchange factors (VAVs), crucial for signal transduction between cell membrane receptors and intracellular mediators, have been implicated in carcinogenesis. However, their potential prognostic value in RCC remains unclear. The impact of 150 common *VAV* polymorphisms on RCC risk and survival was investigated in a cohort of 630 individuals. Publicly available gene expression datasets were utilized to analyze *VAV* gene expression in relation to patient outcomes. The *VAV3* rs17019888 polymorphism was significantly associated with RCC risk and overall survival after adjusting for false discovery rates. Expression quantitative trait loci analysis revealed that the risk allele of rs17019888 is linked to reduced *VAV3* expression. Analysis of 19 kidney cancer gene expression datasets revealed lower *VAV3* expression in RCC tissues compared to normal tissues, with higher expression correlating with better prognosis. Gene set enrichment analysis demonstrated that *VAV3* negatively regulates the ubiquitin–proteasome system, extracellular matrix and membrane receptors, inflammatory responses, matrix metalloproteinases, and cell cycle pathways. Furthermore, elevated *VAV3* expression was associated with increased infiltration of B cells, macrophages, and neutrophils into the RCC tumor microenvironment. Our findings suggest that *VAV3* gene variants influence RCC risk and survival, contributing to a favorable prognosis in RCC.

## 1. Introduction

Renal cell carcinoma (RCC) is among the most prevalent forms of genitourinary cancer, with global incidence rates rising by approximately 2–3% annually [1]. From 1990 to 2019, the number of RCC cases worldwide has increased by 154.78% [2]. The mortality rate of RCC ranges from 30 to 40%, with a higher prevalence in males and in developed countries. The age of onset has shifted to younger populations, likely due to widespread health screening [3]. Despite this, the 5-year survival rate remained significantly lower in metastatic cases (13%) than in regional cases (70%) [4]. Current treatments for advanced unresectable RCC are inadequate, underscoring the urgent need for novel therapeutic strategies. Genetic factors and single-nucleotide polymorphisms (SNPs) such as *VHL* rs7629500 and *IRF5* rs3807306 play significant roles in RCC susceptibility [5]. Therefore, elucidating the genetic and molecular mechanisms underlying RCC may lead to the development of early diagnostic markers and effective treatments.

*VAV* genes function as guanine nucleotide exchange factors (GEFs) that activate Rho family GTPases [6]. In mammals, the *VAV* family comprises *VAV1*, *VAV2*, and *VAV3*, which have similar but not identical functions. *VAV1* is primarily expressed in hematopoietic cells, whereas *VAV2* and *VAV3* exhibit more widespread expression patterns [7]. VAVs play a crucial role in the intracellular signaling pathways downstream of receptors with tyrosine kinase activity [8]. Upon receptor stimulation, VAV proteins undergo tyrosine phosphorylation, activating Rho GTPases such as RhoA, Rac1, and Cdc42 [6], which, in turn, regulate various cellular processes, including phagocytosis, vesicular transport, cell growth, chemotaxis, migration, and adhesion [9].

Recent studies suggest that VAV proteins are essential for maintaining the homeostasis of cardiovascular, central nervous, and immune systems [6]. They are also implicated in numerous aspects of cancer biology, such as epithelial–mesenchymal transition, tumorigenesis, chemosensitivity, and metastasis [10,11]. *VAV1*, initially identified for its oncogenic activity [12], has been associated with poor survival rates in pancreatic [13], ovarian [14], and medulloblastoma tumors [15]. Conversely, *VAV1* may function as a tumor suppressor in immature T cells, with its loss leading to acute lymphoblastic leukemia in T cells [16,17]. Recent studies have investigated the relationship between genetic variants of *VAV* family genes and cancer susceptibility. For example, *VAV3* rs7521681 and rs4915076 have been linked to thyroid cancer risk [18], whereas rs12410676 has been associated with prostate cancer in Chinese males [19]. Additionally, *VAV2* rs12002767 may influence the overall survival of patients with non-small cell lung cancer by regulating *VAV2* expression [20]. However, the clinical significance of the genetic variants of *VAV* family genes in RCC remains unclear.

Given the critical role of *VAV* GEF family genes in signal transduction and cellular behavior, we hypothesized that genetic variants in *VAV* family genes are associated with RCC risk and survival. To test this hypothesis, we examined the relationship between 150 SNPs in *VAV* family genes and RCC risk and survival outcomes in a cohort of 630 participants. Additionally, we explored the functional significance of the implicated gene, *VAV3*, through gene ontology (GO) enrichment and pathway analyses to elucidate the potential biological mechanisms influencing RCC development.

## 2. Materials and Methods

### 2.1. Study Population and Data Collection

This study involved 630 participants recruited from Taipei Medical University Hospital, Taipei Municipal Wan Fang Hospital, and National Taiwan University Hospital. The cohort comprised 312 patients with pathologically confirmed RCC and 318 cancer-free controls matched for age (±1 year) and sex [21,22]. Demographic information was gathered through structured interviews, and clinical data were extracted from medical records. Our patient cohort consisted of 75.3% clear cell RCC (KIRC), 8.7% papillary RCC (KIRP), and 8% chromophobe RCC (KICH), closely mirroring the general cancer statistics where these subtypes represent approximately 75%, 10%, and 5% of all kidney cancers, respectively. As shown in Table S1, the control group and patients with RCC were similar regarding sex, age, body mass index (BMI), and smoking status, but showed significant differences in alcohol intake, hypertension, and diabetes prevalence (*p* < 0.001). Most RCC cases were at stages I–II (81.4%) or grade I–II (75.2%). During the median follow-up period of 90 months, 34 (10.9%) patients died. All participants provided written informed consent prior to the interviews and specimen collection. This study was conducted in compliance with the Declaration of Helsinki and received approval from the Research Ethics Committee of the National Taiwan University Hospital (approval no. 9100201527, 2 July 2012).

### 2.2. SNP Selection and Genotyping

We selected haplotype-tagged SNPs for the *VAV* genes (*VAV1*, *VAV2*, and *VAV3*) using Han Chinese data from the 1000 Genomes Project and Haploview version 4.2, applying the criteria of minor allele frequency >0.03 and pairwise linkage disequilibrium *r*^2^ > 0.8 [23,24]. A total of 150 SNPs was identified and genotyped. Genomic DNA was extracted from peripheral blood using the QIAamp DNA Blood Mini Kit (Qiagen, Valencia, CA, USA). Genotyping was performed using the Affymetrix Axiom Genotyping Arrays at the National Centre for Genome Medicine, Taiwan, in accordance with established protocols [25]. All SNPs were in Hardy–Weinberg equilibrium (*p* > 0.001), with genotyping rates between 97.9 and 100.0%.

### 2.3. Bioinformatic Analyses

Expression quantitative trait loci (eQTL) associations between SNPs and gene expression were evaluated using the Genotype-Tissue Expression (GTEx) portal [26]. Functional predictions of the SNPs were performed using HaploReg [27]. We analyzed 19 kidney cancer gene expression datasets from Gene Expression Omnibus [28,29,30,31,32,33,34,35,36,37,38,39,40,41], ArrayExpress [42,43], and The Cancer Genome Atlas (TCGA) [44] to explore correlations between gene expression and patient prognosis. For a deeper insight into the molecular mechanisms and pathways involving *VAV3*, we analyzed the genes correlated with *VAV3* in TCGA-KIRC kidney renal clear cell carcinoma samples using Pearson’s correlation via LinkedOmics [45]. Enrichment analysis of GO terms and WikiPathways was conducted using gene set enrichment analysis (GSEA), with thresholds set at a false discovery rate (FDR) <0.05 and 1000 simulations. Tumor-infiltrating immune cell infiltration levels in TCGA-KIRC in relation to *VAV3* somatic copy number alterations and expression were compared using the TIMER database [46].

### 2.4. Statistical Analyses

Logistic regression was used to assess SNP associations with RCC risk by calculating odds ratios (ORs) and 95% confidence intervals (CIs) using the Statistical Package for the Social Sciences (version 19.0.0; IBM, Armonk, NY, USA). Statistical significance was defined as *p* < 0.05, and multiple testing corrections were applied using the FDR (*q*-value) [47]. *VAV3* expression levels in cancer and adjacent normal tissues were compared using standardized mean difference (SMD) and 95% CI with a random-effects model in Review Manager (RevMan version 5.4.1; Cochrane, London, UK). Associations between *VAV3* mRNA expression and RCC patient survival were evaluated by pooling hazard ratios (HRs) and 95% CIs using a random-effects model with RevMan.

## 3. Results

Logistic regression analysis was conducted to examine the association between genetic variants in *VAV* genes and the risk of RCC (Table S2). Of the 150 SNPs analyzed, seven SNPs within the *VAV* genes demonstrated a significant association with the risk of RCC (*p* < 0.05). Notably, the most significant association was observed for SNP rs17019888 in the *VAV3* gene, which had a *q*-value of 0.320, suggesting that 32.0% of these associations may be false positives (less than one false discovery). Specifically, each additional minor allele C of *VAV3* rs17019888 was associated with a 33% reduction in RCC risk (OR = 0.67, 95% CI = 0.50–0.88, *p* = 0.005; Table 1). This association remained significant after adjusting for confounding factors including sex, age, BMI, smoking status, alcohol intake, and history of diabetes and hypertension (adjusted OR = 0.59, 95% CI = 0.43–0.81, *p* = 0.001; Table 1).

**Figure 1:**
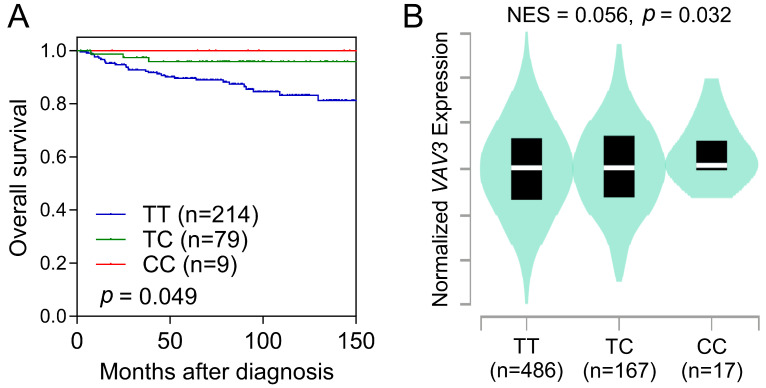
Survival and expression quantitative trait loci analyses for *VAV3* rs17019888. (**A**) Kaplan–Meier estimation displays overall survival of patients with renal cell carcinoma (RCC) categorized based on the rs17019888 genotype of *VAV3*. (**B**) Association between the genotypes of rs17019888 and the expression of *VAV3* in whole blood based on the Genotype-Tissue Expression dataset. NES, normalized effect size.

The minor C allele of *VAV3* rs17019888 was also linked to overall survival among patients with RCC. Individuals carrying the minor allele C had a 68% reduction in the risk of all-cause mortality compared to those with the major allele homozygous genotype (TT) (HR = 0.32, 95% CI = 0.11–0.90, *p* = 0.032; Table 2 and Figure 1A). This association persisted after adjusting for sex, age, BMI, smoking status, alcohol intake, history of diabetes and hypertension, and disease stage and grade (adjusted HR = 0.12, 95% CI = 0.02–0.89, *p* = 0.038; Table 2).

Subsequent eQTL analyses of 670 whole-blood samples from the GTEx project revealed that the minor C allele of rs17019888 was associated with increased *VAV3* mRNA expression (*p* = 0.032; Figure 1B). Despite rs17019888 being located within an intron of *VAV3*, functional prediction using HaploReg indicated that several SNPs in high linkage disequilibrium with rs17019888 coincided with potential promoter or enhancer regions (Table S3).

**Figure 2:**
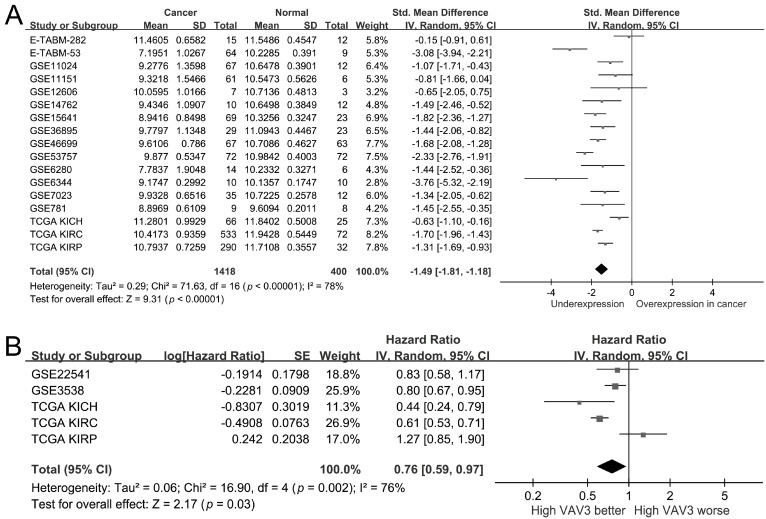
The mRNA expression of *VAV3* in kidney cancer. (**A**) Forest plot of differential expression of *VAV3* in kidney cancer and normal tissues. (**B**) Forest plot showing the association between *VAV3* expression and kidney cancer prognosis. SD, standard deviation; SE, standard error; IV, inverse variance; CI, confidence interval; Std, standardized; TCGA, The Cancer Genome Atlas; KICH, kidney chromophobe; KIRC, kidney renal clear cell carcinoma; KIRP, kidney renal papillary cell carcinoma; df, degrees of freedom [28,29,30,31,32,33,34,35,36,37,38,39,40,41,42,43,44].

To better understand *VAV3*’s role in RCC, public kidney cancer datasets were analyzed. A pooled analysis of 1418 kidney cancer and 400 adjacent normal tissues across 17 studies revealed significant downregulation of *VAV3* in kidney cancer (SMD = −1.49, 95% CI = −1.81 to −1.18, *p* < 0.001; Figure 2A). Moreover, higher *VAV3* expression was significantly linked to improved patient survival (HR = 0.76, 95% CI = 0.59–0.97, *p* = 0.03; Figure 2B). Interestingly, *VAV3* exhibited downregulation (SMD from −0.63 to −1.70; Figure 2A) across all three TCGA datasets for the main RCC subtypes: KIRC, KIRP, and KICH. High *VAV3* expression was significantly associated with improved survival in the TCGA KIRC and KICH datasets, while it tended to be linked to poor survival in the KIRP dataset (Figure 2B).

**Figure 3:**
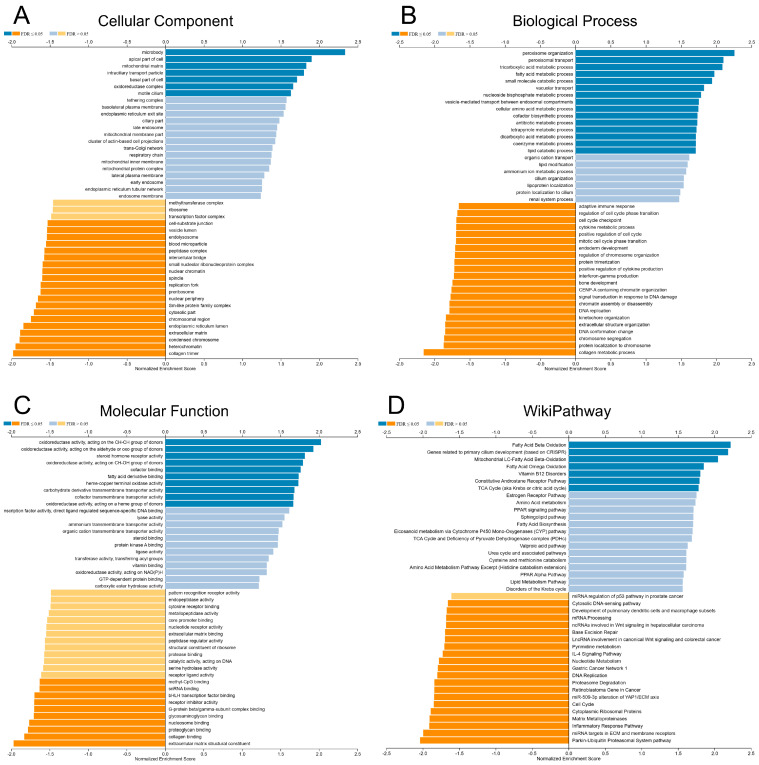
Potential biological functions of *VAV3* in kidney cancer. Gene ontology annotations of (**A**) cellular components, (**B**) biological processes, (**C**) molecular functions, and (**D**) WikiPathway enrichment analysis of *VAV3* correlated genes.

To understand the biological significance of *VAV3* in RCC, we investigated *VAV3*-correlated genes in the TCGA-KIRC cohort. We identified 3298 positively correlated and 5606 negatively correlated genes based on FDR < 0.01 for Pearson’s correlation. GO term annotation indicated that *VAV3*-correlated genes were involved in cellular components such as the collagen trimer, condensed chromatin, and extracellular matrix (Figure 3A). These genes were primarily involved in biological processes such as collagen metabolic processes, chromosome segregation, and extracellular structure organization (Figure 3B). Molecular functions, such as extracellular matrix structural constituents, collagen binding, and nucleosome binding, were negatively regulated by *VAV3* (Figure 3C). WikiPathway enrichment analysis suggested that *VAV3* negatively regulates pathways related to the ubiquitin–proteasome system, extracellular matrix and membrane receptors, inflammatory response, matrix metalloproteinases, and cell cycle (Figure 3D), which indicate a protective role of *VAV3* against RCC.

**Figure 4:**
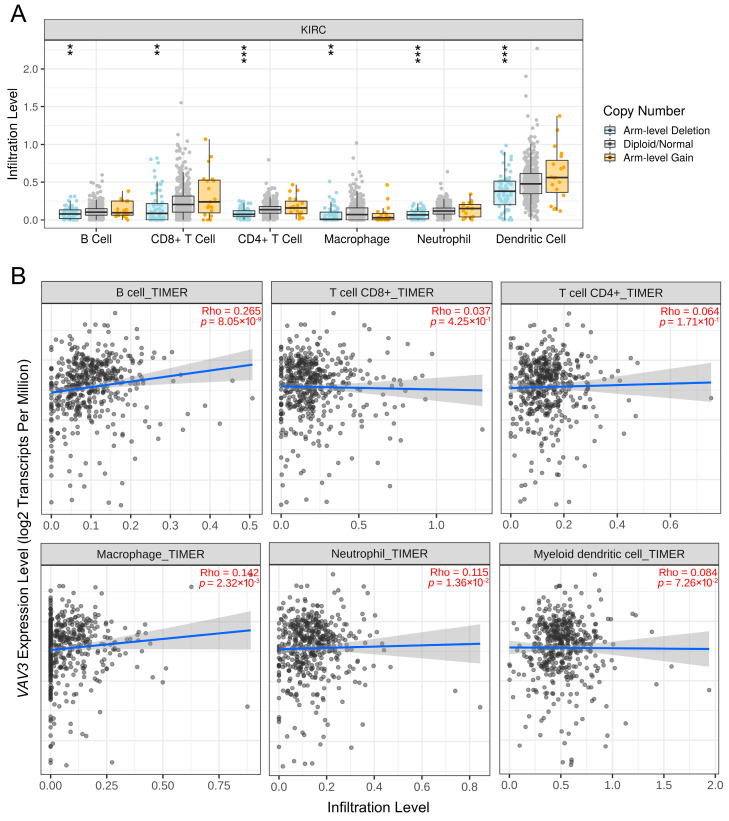
Correlation between *VAV3* expression and immune cell infiltration levels in the tumor microenvironment. (**A**) *VAV3* copy number variations affect the infiltration levels of multiple immune cell types in The Cancer Genome Atlas kidney renal clear cell carcinoma dataset. ** *p* < 0.01; *** *p* < 0.001. (**B**) Correlation between *VAV3* expression and the infiltrating levels of B cells, CD8^+^ T cells, CD4^+^ T cells, macrophages, neutrophils, and dendritic cells.

Given the association between the inflammatory response and cancer development, we examined the correlation between *VAV3* expression and immune cell infiltration in RCC. Arm-level deletion of *VAV3* was associated with the infiltration of various immune cells, including B cells, CD8^+^ T cells, CD4^+^ T cells, macrophages, neutrophils, and dendritic cells (Figure 4A). Furthermore, the infiltration levels of B cells, macrophages, and neutrophils positively correlated with *VAV3* expression in RCC (Figure 4B). These findings suggest that *VAV3* plays a crucial role in modulating immune cell infiltration, particularly in B cells, macrophages, and neutrophils, in RCC.

## 4. Discussion

In this study, we employed a combination of genetic and bioinformatics analyses to investigate the associations between genetic variants in *VAV* family genes and the risk and survival of RCC thoroughly. We identified a significant association between the *VAV3* rs17019888 T > C polymorphism and the risk and overall survival of patients with RCC, which remained robust after multivariate analysis and multiple testing correction. Carriers of the rs17019888 C allele exhibited a reduced risk of developing RCC and lower rates of all-cause mortality. Additionally, the rs17019888 C allele was significantly correlated with increased *VAV3* mRNA expression. Our pooled analysis of 19 studies indicated that *VAV3* may have tumor-suppressive properties, as it is downregulated in kidney cancers, and that higher *VAV3* mRNA expression levels were associated with better survival outcomes in patients with RCC. GSEA further suggested that *VAV3* may negatively regulate pathways related to the ubiquitin–proteasome system, extracellular matrix and membrane receptors, inflammatory response, matrix metalloproteinases, and cell cycle. Higher *VAV3* expression was also correlated with increased infiltration of anticancer immune cells, which is consistent with our genetic findings.

The VAV family of proteins, known as signal transducers, was initially identified for their protumorigenic activities. Various studies have reported that *VAV3* enhances cell proliferation in cancers such as breast cancer [11], gastric cancer [48], endometrial cancer [49], osteosarcoma [50], and acute lymphoblastic leukemia [51]. In addition, *VAV3* promotes cell migration and invasion in breast cancer [52], pancreatic cancer [53], gastric cancer [48], and osteosarcoma [50]. These findings highlight *VAV3* as the pivotal player in cancer progression. However, despite its oncogenic effects and upregulation in several tumors, *VAV3* expression is downregulated in certain cancers, including colon adenocarcinoma, head and neck squamous cell carcinoma, kidney cancer, lung adenocarcinoma, and prostate adenocarcinoma, as indicated by TCGA datasets. These data suggest that *VAV3* expression levels and their prognostic significance are highly cancer-dependent, necessitating further investigations to confirm *VAV3*’s specific role in different cancer types.

Furthermore, recent studies have shown that *VAV1*-deficient mice develop T-cell tumors at high frequencies upon aging or carcinogen treatment [17,54]. *VAV1* silences NOTCH1 signaling and suppresses T cell acute lymphoblastic leukemia by promoting the degradation of the intracellular domain of NOTCH1 (ICN1) through ubiquitination and proteasomal degradation [17]. Given the structural similarity and overlapping functions of VAV proteins, it is plausible that *VAV3* may also play tumor suppressor roles akin to *VAV1*. Studies have reported that *VAV3*-deficient mice develop certain tumors more frequently than their wild-type counterparts [55].

This study is the first to explore the clinical significance and role of *VAV3* in RCC using bioinformatic and functional analyses. Our pooled analysis of multiple datasets demonstrated that *VAV3* expression was reduced in RCC tumors and that patients with higher *VAV3* expression had longer survival times. GSEA revealed that genes co-expressed with *VAV3* were enriched in pathways related to the ubiquitin–proteasomal system, extracellular matrix and membrane receptors, inflammatory response, matrix metalloproteinases, and cell cycle. Additionally, high *VAV3* expression was correlated with increased infiltration of anticancer immune cells. These findings suggested that *VAV3* plays a suppressive role in RCC, possibly through ubiquitin-mediated proteasomal degradation, thereby influencing cancer-associated pathways. However, further research is required to elucidate the precise molecular mechanisms underlying *VAV3*’s involvement in RCC.

Despite these significant findings, this study has several limitations. First, the small sample size may have reduced the statistical power of detecting significant associations. Second, all study participants were Taiwanese, which may limit the generalizability of our findings to other ethnicities. Third, the lack of detailed clinical information from public datasets precluded adjustments to our analyses. Finally, the exact molecular mechanisms underlying the associations between the identified SNP and RCC survival remain unclear and require further investigation.

## 5. Conclusions

Our findings suggest that *VAV3* rs17019888 T > C polymorphism may serve as a novel prognostic biomarker for RCC. The rs17019888 variant may influence RCC progression by modulating *VAV3* expression and affecting cancer-related pathways and immune cell infiltration. However, it is essential to validate these results in larger studies and to conduct further mechanistic investigations to gain a comprehensive understanding of the underlying mechanisms.

## Figures and Tables

**Table 1:** Association of *VAV3* rs17019888 with the risk of renal cell carcinoma.

Genotype	Controls, *n* (%)	Patients, *n* (%)	OR (95% CI)	*p*	*q*	OR (95% CI) ^a^	*p* ^a^
TT	191 (60.8)	214 (70.9)	1.00			1.00	
TC	104 (33.1)	79 (26.2)	0.68 (0.48–0.96)	0.030		0.56 (0.38–0.82)	0.003
CC	19 (6.1)	9 (3.0)	0.42 (0.19–0.96)	0.039		0.42 (0.17–1.05)	0.064
TC/CC			0.64 (0.46–0.89)	0.009		0.54 (0.37–0.78)	0.001
Trend			0.67 (0.50–0.88)	0.005	0.320	0.59 (0.43–0.81)	0.001

Abbreviations: OR, odds ratio; CI, confidence interval. ^a^ ORs were adjusted for sex, age, body mass index, smoking status, alcohol intake, and history of diabetes and hypertension.

**Table 2:** Association of *VAV3* rs17019888 with overall survival in patients with renal cell carcinoma.

Genotype	*n* of Patients	*n* of Events	5-y Survival Rate (%)	HR (95% CI)	*p*	HR (95% CI) ^a^	*p* ^a^
TT	214	30	89.6	1.00		1.00	
TC	79	3	95.9	0.27 (0.08–0.88)	0.029		
CC	9	1	100.0	0.74 (0.10–5.43)	0.767		
TC/CC	88	4	96.3	0.32 (0.11–0.90)	0.032	0.12 (0.02–0.89)	0.038
Trend				0.42 (0.17–1.03)	0.057	0.28 (0.07–1.08)	0.064

Abbreviations: HR, hazard ratio; CI, confidence interval. ^a^ HRs were adjusted for sex, age, body mass index, smoking status, alcohol intake, history of diabetes and hypertension, and disease stage and grade.

## Data Availability

Data will be available on reasonable request.

## Supplementary Materials

The following supporting information can be downloaded at https://www.mdpi.com/article/10.3390/biomedicines12081694/s1, Table S1. Clinical and demographic characteristics of healthy controls and patients with renal cell carcinoma. Table S2. Association between vav guanine nucleotide exchange factor gene polymorphisms and the risk of renal cell carcinoma. Table S3. Regulatory annotation of *VAV3* rs17019888.
