# Supplementary material for: Prognostic Significance of VAV3 Gene Variants and Expression in Renal Cell Carcinoma
Source: Biomedicines. 2024 Jul 30;12(8):1694. doi: 10.3390/biomedicines12081694 (PMC11351164; doi:10.3390/biomedicines12081694)
Supplement: Supplementary file 1 [file biomedicines-12-01694-s001.zip › biomedicines-3117375-supplementary.pdf]

Table S1. Clinical and demographic characteristics of healthy controls and patients with renal cell carcinoma

Characteristic	Controls (n = 318)	Patients (n = 312)	*P*
Sex, n (%)			
Male	210 (66.0)	209 (67.0)	0.801
Female	108 (34.0)	103 (33.0)	
Age, y			
Median (IQR)	57 (47-68)	57 (48-68)	
≤57	162 (50.9)	160 (51.3)	0.932
>57	156 (49.1)	152 (48.7)	
Body mass index, kg/m^2			
Median (IQR)	24.6 (22.6-26.7)	24.4 (22.3-27.6)	
≤25	163 (57.0)	172 (55.5)	0.711
>25	123 (43.0)	138 (44.5)	
Alcohol intake, n (%)			
Ever	135 (42.5)	76 (24.4)	<0.001
Never	183 (57.5)	236 (75.6)	
Smoking status, n (%)			
Ever	105 (33.0)	114 (36.5)	0.354
Never	213 (67.0)	198 (63.5)	
Hypertension, n (%)			
Yes	78 (24.9)	134 (43.1)	<0.001
No	235 (75.1)	177 (56.9)	
Diabetes, n (%)			
Yes	20 (6.3)	62 (19.9)	<0.001
No	296 (93.7)	249 (80.1)	

Stage, n (%)	
I-II	240 (81.4)
III-IV	55 (18.6)
Grade, n (%)	
I-II	206 (75.2)
III-IV	68 (24.8)
Survival status[a]	
Alive	278 (89.1)
Death	34 (10.9)

Abbreviations: IQR, interquartile range.

[a]With a median follow-up of 90 months.

Subtotals do not sum to n of patients due to missing data.

Table S2. Association between vav guanine nucleotide exchange factor gene polymorphisms and the risk of renal cell carcinoma

Gene	SNP ID	Chromosome	Position	Allele	MAF	HWE	*P*	*q*
VAV3	rs12124854	1	107569437	C>T	0.042	0.106	0.545	0.459
VAV3	rs11185141	1	107590257	T>C	0.409	0.377	0.467	0.435
VAV3	rs1536664	1	107609235	T>C	0.346	0.391	0.795	0.483
VAV3	rs56329640	1	107622298	G>A	0.059	0.158	0.929	0.509
VAV3	rs10465781	1	107645478	C>T	0.178	0.423	0.991	0.529
VAV3	rs17019602	1	107646236	A>G	0.168	0.890	0.395	0.424
VAV3	rs150148423	1	107646801	G>A	0.037	0.604	0.413	0.429
VAV3	rs80142139	1	107654097	G>A	0.121	1.000	0.456	0.435
VAV3	rs10494071	1	107660257	C>T	0.454	0.005	0.523	0.454
VAV3	rs117505667	1	107663063	G>A	0.034	0.540	0.185	0.346
VAV3	rs2494051	1	107676029	G>A	0.377	0.934	0.747	0.483
VAV3	rs11811342	1	107679713	C>A	0.156	0.103	0.606	0.462
VAV3	rs12132327	1	107685712	G>T	0.239	0.520	0.154	0.346
VAV3	rs12039221	1	107693582	G>C	0.139	0.144	0.448	0.432
VAV3	rs72705616	1	107694238	T>C	0.481	0.277	0.716	0.483
VAV3	rs12040706	1	107697539	T>C	0.215	0.908	0.056	0.346
VAV3	rs146720455	1	107698486	T>C	0.036	1.000	0.709	0.483
VAV3	rs12043814	1	107701229	C>T	0.480	0.816	0.339	0.424
VAV3	rs2504468	1	107702634	T>C	0.333	0.136	0.304	0.424
VAV3	rs17019729	1	107703042	T>C	0.318	0.019	0.834	0.494
VAV3	rs138817273	1	107708867	C>T	0.032	1.000	0.789	0.483
VAV3	rs77841908	1	107713714	C>T	0.073	0.566	0.397	0.424
VAV3	rs144733602	1	107725437	G>A	0.032	0.506	0.797	0.483

VAV3	rs17019855	1	107742766	A>G	0.452	0.754	0.886	0.496
VAV3	rs17019888	1	107747630	T>C	0.194	0.257	0.005	0.320
VAV3	rs12037244	1	107751599	C>G	0.347	0.304	0.329	0.424
VAV3	rs12138353	1	107757187	G>A	0.321	0.152	0.766	0.483
VAV3	rs150611337	1	107762252	C>T	0.038	0.618	0.208	0.358
VAV3	rs6583048	1	107770636	T>C	0.332	0.380	0.079	0.346
VAV3	rs189590560	1	107770841	C>T	0.033	0.508	0.480	0.437
VAV3	rs79537343	1	107786080	T>C	0.031	0.473	0.708	0.483
VAV3	rs4914961	1	107791141	C>T	0.244	0.461	0.066	0.346
VAV3	rs12217032	1	107807704	A>G	0.116	0.444	0.154	0.346
VAV3	rs114937543	1	107809084	G>A	0.051	0.079	0.295	0.424
VAV3	rs6703923	1	107809630	A>G	0.300	0.926	0.893	0.496
VAV3	rs12126655	1	107814198	A>G	0.308	0.465	0.158	0.346
VAV3	rs4915077	1	107823394	T>C	0.251	0.352	0.408	0.429
VAV3	rs13373947	1	107855772	A>G	0.076	0.045	0.600	0.462
VAV3	rs1075564	1	107865371	A>G	0.165	0.779	0.176	0.346
VAV3	rs1999487	1	107866361	T>C	0.315	0.071	0.420	0.431
VAV3	rs1999486	1	107866416	G>T	0.403	0.043	0.757	0.483
VAV3	rs12029201	1	107866458	A>G	0.405	0.333	0.056	0.346
VAV3	rs12750602	1	107875677	T>C	0.384	1.000	0.636	0.476
VAV3	rs11185193	1	107884051	G>A	0.036	0.572	0.144	0.346
VAV3	rs17020273	1	107887673	T>C	0.094	0.354	0.076	0.346
VAV3	rs17020276	1	107894467	C>T	0.051	0.688	0.751	0.483
VAV3	rs17020294	1	107899020	T>C	0.377	1.000	0.442	0.431
VAV3	rs77149713	1	107902633	T>G	0.057	0.001	0.475	0.437

VAV3	rs345299	1	107905511	C>A	0.247	0.143	0.392	0.424
VAV3	rs6685719	1	107913363	C>T	0.067	0.200	0.901	0.497
VAV3	rs79597802	1	107932723	G>T	0.086	1.000	0.239	0.371
VAV3	rs77088232	1	107936809	A>C	0.047	0.648	0.008	0.320
VAV3	rs17020437	1	107941495	T>C	0.064	0.181	0.568	0.459
VAV3	rs9435347	1	107941804	T>C	0.291	0.023	0.765	0.483
VAV3	rs75317139	1	107956897	G>A	0.036	1.000	0.320	0.424
VAV3	rs117107124	1	107967464	C>T	0.055	0.434	0.771	0.483
VAV2	rs480809	9	133754156	G>A	0.239	0.163	0.330	0.424
VAV2	rs2502763	9	133755625	C>A	0.264	0.764	0.179	0.346
VAV2	rs35237364	9	133756566	T>G	0.123	0.718	0.376	0.424
VAV2	rs591797	9	133765682	G>A	0.039	1.000	0.606	0.462
VAV2	rs622035	9	133767540	C>A	0.464	0.050	0.393	0.424
VAV2	rs656702	9	133771439	C>A	0.109	0.552	0.678	0.483
VAV2	rs2106373	9	133786837	A>G	0.420	0.576	0.861	0.496
VAV2	rs2073888	9	133793694	T>C	0.455	0.240	0.979	0.529
VAV2	rs79643046	9	133804529	C>T	0.055	1.000	0.566	0.459
VAV2	rs76605198	9	133807606	C>A	0.056	0.444	0.338	0.424
VAV2	rs2428091	9	133808563	G>T	0.261	0.614	0.442	0.431
VAV2	rs756777	9	133813958	A>G	0.080	1.000	0.467	0.435
VAV2	rs59778917	9	133817931	G>A	0.056	0.450	0.738	0.483
VAV2	rs3893493	9	133828784	G>A	0.077	0.788	0.883	0.496
VAV2	rs76518601	9	133830254	G>A	0.167	0.049	0.184	0.346
VAV2	rs60802618	9	133835483	T>C	0.408	0.147	0.111	0.346
VAV2	rs2428123	9	133848874	T>C	0.125	0.213	0.168	0.346

VAV2	rs117374863	9	133852055	G>T	0.036	1.000	0.384	0.424
VAV2	rs1150063	9	133852609	T>G	0.048	0.179	0.227	0.371
VAV2	rs140158130	9	133868256	C>T	0.036	0.049	0.062	0.346
VAV2	rs62576553	9	133873207	A>G	0.039	0.265	0.148	0.346
VAV2	rs12555091	9	133873212	G>A	0.139	0.419	0.156	0.346
VAV2	rs3780741	9	133874964	G>A	0.055	0.042	0.759	0.483
VAV2	rs10739995	9	133876227	A>G	0.072	0.372	0.656	0.483
VAV2	rs2486354	9	133889071	C>T	0.179	0.595	0.732	0.483
VAV2	rs117957098	9	133889814	T>C	0.033	1.000	0.384	0.424
VAV2	rs2519768	9	133890636	T>C	0.123	0.468	0.210	0.358
VAV2	rs13291383	9	133897690	T>C	0.310	0.716	0.854	0.495
VAV2	rs2810493	9	133902013	C>T	0.062	0.002	0.158	0.346
VAV2	rs4744525	9	133904602	C>A	0.036	1.000	0.573	0.459
VAV2	rs679923	9	133906310	A>G	0.323	1.000	0.157	0.346
VAV2	rs745695	9	133911151	T>C	0.318	0.420	0.383	0.424
VAV2	rs608402	9	133914031	A>G	0.262	0.615	0.121	0.346
VAV2	rs583505	9	133915383	C>T	0.344	0.047	0.812	0.489
VAV2	rs10116423	9	133915428	C>T	0.050	1.000	0.083	0.346
VAV2	rs624234	9	133921802	T>C	0.424	0.339	0.527	0.454
VAV2	rs2265520	9	133925160	C>T	0.331	0.792	0.887	0.496
VAV2	rs2492056	9	133925526	T>G	0.047	0.390	0.387	0.424
VAV2	rs2519100	9	133936650	C>G	0.367	1.000	0.186	0.346
VAV2	rs2519109	9	133942648	G>A	0.169	0.094	0.659	0.483
VAV2	rs503596	9	133945402	G>A	0.356	0.799	0.049	0.346
VAV2	rs687644	9	133947212	A>G	0.446	0.814	0.170	0.346

VAV2	rs3780783	9	133948246	C>A	0.189	0.126	0.725	0.483
VAV2	rs493433	9	133950868	A>G	0.428	0.475	0.216	0.360
VAV2	rs524538	9	133951976	A>C	0.390	0.807	0.090	0.346
VAV2	rs654182	9	133952064	T>C	0.415	0.873	0.557	0.459
VAV2	rs2519796	9	133960211	A>G	0.209	0.479	0.132	0.346
VAV2	rs10119727	9	133963246	G>A	0.229	1.000	0.145	0.346
VAV2	rs2810539	9	133966102	C>T	0.393	0.416	0.951	0.518
VAV2	rs452943	9	133967455	C>T	0.395	0.516	0.533	0.454
VAV2	rs3780792	9	133970221	A>G	0.121	0.713	0.205	0.358
VAV2	rs438210	9	133970364	C>T	0.293	0.707	0.286	0.424
VAV2	rs2810542	9	133970538	C>G	0.067	1.000	0.695	0.483
VAV2	rs62577870	9	133971024	A>G	0.287	0.254	0.304	0.424
VAV2	rs679547	9	133974611	T>G	0.144	0.428	0.197	0.358
VAV2	rs77221761	9	133977524	G>T	0.081	1.000	0.151	0.346
VAV2	rs431791	9	133986152	A>G	0.158	1.000	0.079	0.346
VAV2	rs459797	9	133997407	G>A	0.409	0.295	0.623	0.470
VAV2	rs461566	9	134000238	T>G	0.106	0.836	0.071	0.346
VAV1	rs74574985	19	6766737	A>C	0.087	0.323	0.241	0.371
VAV1	rs10413156	19	6769404	T>C	0.069	0.536	0.111	0.346
VAV1	rs682626	19	6774058	G>A	0.173	0.172	0.873	0.496
VAV1	rs62125158	19	6775899	G>A	0.298	0.401	0.425	0.431
VAV1	rs2660478	19	6783342	T>C	0.127	0.600	0.137	0.346
VAV1	rs2617819	19	6796369	G>C	0.166	0.888	0.699	0.483
VAV1	rs11085201	19	6796711	A>G	0.097	0.186	0.559	0.459
VAV1	rs4807901	19	6796723	T>A	0.069	0.226	0.503	0.442

VAV1	rs2617822	19	6801470	A>G	0.067	0.350	0.847	0.495
VAV1	rs12610223	19	6808645	A>G	0.187	0.021	0.370	0.424
VAV1	rs11085204	19	6808751	C>T	0.120	0.138	0.346	0.424
VAV1	rs374119	19	6812091	A>G	0.084	0.800	0.839	0.494
VAV1	rs73484417	19	6813606	C>G	0.048	0.178	0.234	0.371
VAV1	rs10419572	19	6814549	T>A	0.204	0.472	0.169	0.346
VAV1	rs4807100	19	6815713	G>A	0.301	0.310	0.772	0.483
VAV1	rs77953201	19	6817481	T>G	0.135	0.868	0.792	0.483
VAV1	rs58882129	19	6818559	C>T	0.351	0.201	0.606	0.462
VAV1	rs10415535	19	6818690	G>A	0.093	0.351	0.991	0.529
VAV1	rs56333516	19	6818726	G>A	0.045	1.000	0.365	0.424
VAV1	rs164016	19	6820940	T>C	0.465	0.184	0.435	0.431
VAV1	rs7258040	19	6821534	C>T	0.068	0.760	0.019	0.346
VAV1	rs28617395	19	6821614	C>T	0.047	1.000	0.710	0.483
VAV1	rs347033	19	6822208	T>C	0.243	0.832	0.012	0.320
VAV1	rs2288545	19	6825296	C>T	0.151	0.361	0.606	0.462
VAV1	rs79352536	19	6825687	A>G	0.042	0.624	0.178	0.346
VAV1	rs164022	19	6828029	G>C	0.340	1.000	0.502	0.442
VAV1	rs3786688	19	6831644	G>A	0.156	0.768	0.489	0.440
VAV1	rs117857922	19	6832844	G>A	0.033	1.000	0.697	0.483
VAV1	rs10422749	19	6833059	A>C	0.057	0.716	0.835	0.494
VAV1	rs308196	19	6836260	C>A	0.261	0.157	0.048	0.346
VAV1	rs60930171	19	6838678	C>T	0.067	0.350	0.366	0.424
VAV1	rs331681	19	6844934	C>T	0.258	0.310	0.039	0.346
VAV1	rs331679	19	6848209	G>A	0.386	0.219	0.086	0.346

VAV1	rs461970	19	6857629	C>T	0.374	1.000	0.123	0.346
VAV1	rs17718517	19	6862487	T>C	0.314	0.588	0.306	0.424

Abbreviations: SNP, single nucleotide polymorphism; MAF, minor alleles frequency; HWE, Hardy-Weinberg equilibrium.

Table S3. Regulatory annotation of *VAV3* rs17019888

Position	SNP ID	LD (r^2)	Reference allele	Alternate allele	ASN frequency	Promoter histone marks	Enhancer histone marks	DNAse	Proteins bound	Motifs changed	eQTL hits
107704391	rs4526642	0.81	G	A	0.23	BRST	STRM, BRST, MUS, CRVX, SKIN			Hoxa9	1 hit
107706157	rs4915067	0.84	C	T	0.23	PANC				AhR::Arnt, Arnt, NRSF	1 hit
107707980	rs6701083	0.84	T	C	0.23					AP-1, CIZ, Nkx3, p300	1 hit
107714909	rs4914956	0.81	A	T	0.24		SKIN			CEBPB, Foxp1, PLZF, Pou1f1, Pou3f2	
107718497	rs4914957	0.86	C	A	0.23					AIRE, p300	1 hit
107724141	rs12022065	0.87	A	G	0.23					Dobox4	1 hit
107724472	rs4276945	0.81	T	C	0.24		FAT				
107728232	rs12029299	0.87	G	A	0.23		PANC			Mef2	
107728453	rs12751558	0.87	A	T	0.23					HNF1, Hoxd8, OTX, Obox6, Pax-4, Pou2f2	
107734959	rs149167681	0.92	A	G	0.22					BCL, Irf, Nkx2, PU.1, Pax-5, RXRA, STAT	
107738238	rs4915070	0.92	A	T	0.22		BLD, SKIN			FXR	

107739098	rs7519428	0.92	G	A	0.22	BLD	BLD, SKIN, LNG, GI	BLD,SKIN	ERALPHA_A, FOXA1,GATA3,P300	Hdx, Pou2f2	1 hit
107740244	rs7553163	0.92	G	A	0.22		BLD, SKIN, LNG, PLCNT, GI				
107742231	rs7547049	0.85	A	G	0.23		GI				1 hit
107743746	rs12031375	0.97	A	G	0.23					Brachyury, E2F	1 hit
107744134	rs6583044	0.94	G	A	0.22		ESDR, LNG			CTCF, Ets, GR	
107746032	rs6695195	0.92	C	T	0.23					AP-1, HDAC2, Nrf-2, TCF11::MafG	
107746825	rs55913072	0.94	A	G	0.24					DMRT1, DMRT7, ERalpha-a	
107747341	rs6668041	1	T	C	0.23						1 hit
107747630	rs17019888	1	T	C	0.23						
107747759	rs35790538	0.95	C	A	0.22					CCNT2, CTCF, Hoxc9, LBP-9	
107748431	rs17019891	0.97	T	C	0.23			LNG		Irf, Pax-5	
107751252	rs7516071	0.94	A	G	0.23					BCL, FAC1, Foxa, Foxj1, Foxj2,	

						Foxk1, Foxl1, Foxo, Foxp1, HDAC2, Irf, Pax-5, Pou2f2, Pou5f1, RXRA, STAT, Sox, Zfp105	
107751277	rs7518423	0.94	T	A	0.23	Cdc5, Foxa, Foxd1, HDAC2, TCF12	1 hit
